# Long-Term Autoimmune Risk Following Pediatric Acute Pyelonephritis: A Nationwide Cohort Study

**DOI:** 10.3390/pathogens15101096

**Published:** 2026-10-21

**Authors:** Young Dong Yu, Dong Soo Park, Man Yong Han

**Affiliations:** 1 Department of Urology, Bundang CHA Hospital, College of Medicine, CHA Medical University, 9 Yatap-ro Bundang-gu, Seongnam-si 13496, Gyeonggi-do, Republic of Korea; danielyu0714@gmail.com; 2 Department of Pediatrics, Bundang CHA Hospital, College of Medicine, CHA Medical University, 9 Yatap-ro Bundang-gu, Seongnam-si 13496, Gyeonggi-do, Republic of Korea

**Keywords:** children, acute pyelonephritis, urinary tract infection, autoimmune diseases, cohort study

## Abstract

Introduction: Acute pyelonephritis (APN) is a serious bacterial infection requiring hospitalization during childhood. This study investigated whether hospitalization for APN is associated with increased subsequent autoimmune disease risk. Methods: In this nationwide, population-based retrospective cohort study using the Korean National Health Insurance Service database, we included children born between 2002 and 2005. Children hospitalized for APN (identified by inpatient ICD-10 codes) were matched by birth year and sex to controls without APN. Clinical data including microbiological outcomes were unavailable. The primary outcome was incident autoimmune disease after a 1-year lag period. Results: The study included 31,856 children with APN and 318,560 matched controls. Autoimmune disease incidence was 98.35 and 59.91 per 10,000 person-years, respectively. APN was associated with increased risk, with heterogeneity across diseases; the strongest association was for IgA nephropathy (aHR 2.14; 95% CI 1.92–2.29). Risks were elevated for autoimmune thyroiditis, Behçet disease, and acute disseminated encephalomyelitis. Recurrent APN carried higher risk than a single episode. Conclusions: Hospitalization for childhood APN was associated with increased autoimmune disease risk through early adolescence. The stronger association with recurrence suggests a dose–response relationship. However, exposure misclassification, unmeasured urinary tract anomalies, and antibiotic-related microbiome effects limit causal inference.

## 1. Introduction

Urinary tract infections (UTIs) are one of the most common and critical bacterial infections in childhood. About 8.4% of girls and 1.7% of boys will have a UTI by seven years of age, whereas the peak incidence of UTI is observed during infancy [1]. Acute pyelonephritis (APN), an acute form of UTI, often presents in the first year of life with high fever and can lead to systemic inflammation and hospitalization, whereas neonates and very young infant stay afebrile in some cases [2]. Severe disruption of immune development period such as infections in early life can interfere the immune system and have been hypothesized to be associated with the risk of autoimmune diseases later in life [3]. Typical examples of infection-triggered autoimmunity include rheumatic fever induced post-group A streptococcus infections as well as post-streptococcal glomerulonephritis (PSGN), or Guillain–Barré syndrome (GBS) associated with preceding *Campylobacter jejuni* infections. The pathogenesis of rheumatic fever and GBS are based on the molecular mimicry between antecedent infectious pathogen and host tissue, whereas PSGN is mediated by immune complexes deposition in the glomeruli [4,5]. Recurrent *Escherichia coli* (*E. coli*)-induced UTIs have also been associated with primary biliary cirrhosis in adults, suggesting that infection may contribute to autoimmunity through molecular mimicry between *E. coli* antigens and mitochondrial PDC-E2 autoantigens [6].

However, definite evidence linking pediatric infections to subsequent autoimmune disorders still remains limited. In addition, some studies postulated that early infections may reduce autoimmune and allergic diseases through the “hygiene hypothesis”, by promoting regulatory immune pathways and immune tolerance [7]. A Swedish cohort study even demonstrated that adults with stress-related disorders had about a 36% greater risk of autoimmune diseases compared to the general population [8]. This emphasizes that external triggers, including psychological stress, may increase the risk of autoimmunity. Therefore, these competing theories highlight the importance to clarify the long-term autoimmune sequelae of serious pediatric infections, although few studies have researched this topic. Another previous study showed that Korean children with *E. coli* infection had a higher incidence of autoimmune diseases than unexposed children [9]. This study highlighted increased risks for specific autoimmune diseases such as systemic lupus erythematosus (SLE), reactive arthritis, and Henoch–Schönlein purpura following *E. coli* infection [9]. APN often requires inpatient treatment, thus provides a unique clinical model to evaluate infection-autoimmunity links. To our knowledge, no prior large-scale study has focused on children with APN to assess their future risk of developing autoimmune diseases.

Therefore, this study conducted a nationwide population-based cohort study to investigate whether children hospitalized for APN have an increased risk of autoimmune diseases during childhood and adolescence. Using Korea’s national health database, this study compared a large cohort of children with APN to infection-free controls to track 42 autoimmune conditions. The study also evaluated for biases and confounding factors like age and sex, including a lag-time analysis to prevent reverse causality.

## 2. Materials and Methods

### 2.1. Study Design and Data Source

This study conducted a nationwide population-based retrospective cohort study using the Korean NHIS database. The NHIS is a mandatory universal health insurance system that provides healthcare coverage for approximately 97% of the Korean population, while the remaining population is covered through the Medical Aid Program. The database contains comprehensive information on demographic characteristics, healthcare utilization, inpatient and outpatient diagnoses, prescription records, procedures, and hospital admissions.

Children born between 1 January 2002, and 31 December 2005, were identified and followed until 31 December 2017. This study was approved by the relevant institutional review board. Because the NHIS database contains de-identified information, informed consent was waived.

### 2.2. Study Population

Children hospitalized for APN were identified using inpatient ICD-10 diagnostic coding and hospitalization records. To improve diagnostic specificity, APN cases were restricted to inpatient admissions with APN-related diagnostic codes rather than outpatient urinary tract infection diagnoses. However, the NHIS claims database did not provide urine culture results, causative organisms, antimicrobial susceptibility, urinalysis, inflammatory markers, or imaging findings (Table S1). Therefore, APN could not be microbiologically classified as *E. coli*, other bacterial infection, or mixed infection. The first hospitalization for APN was designated as the index event. Children with multiple admissions were classified according to recurrence status.

**Figure 1:**
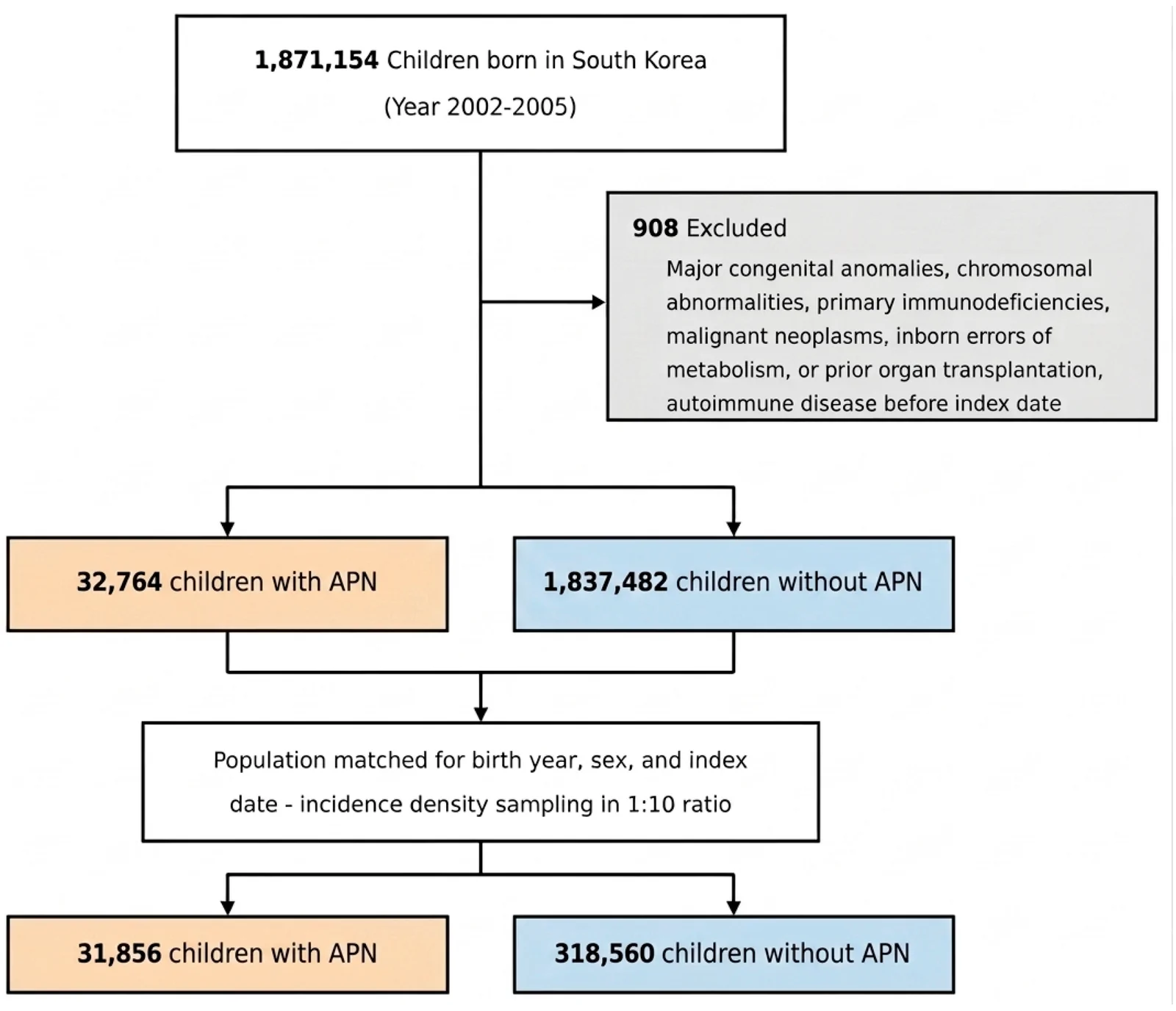
**Flowchart of study subject selection.** Children born between 1 January 2002, and 31 December 2005, were identified and followed until 31 December 2017.

To reduce potential confounding, we excluded children with conditions that could substantially alter immune function or predispose to autoimmune disease development, including major congenital/chromosomal anomalies, primary immunodeficiency disorders, malignancies, and inborn metabolic errors (Figure 1). Children with autoimmune diseases diagnosed before the index date were also excluded. After exclusion of 908 individuals from the 1,871,154 children born during 2002–2005, 32,764 children had an APN hospitalization and 1,837,482 had no APN hospitalization. From these groups, 31,856 children with APN were selected and matched to 318,560 children without APN using incidence-density sampling in a 1:10 ratio according to birth year, sex, and index date. Controls were assigned the same index date as their matched APN case to ensure comparable follow-up periods. The congenital-malformation covariate was retained for subgroup adjustment because the exclusion criterion concerned major congenital/chromosomal anomalies; minor or non-exclusionary malformations could therefore remain in the analytic cohort.

### 2.3. Outcome Definition

The primary outcome was the development of any autoimmune disease after the index date. Because autoimmune diseases may remain undiagnosed during the early stages of clinical presentation, a one-year lag period was applied. Autoimmune diseases diagnosed during the first year following APN were therefore excluded from the primary analysis. Sensitivity analyses used 0-, 2-, 3-, and 5-year lag periods to evaluate reverse causation and diagnostic misclassification.

Autoimmune diseases were identified using predefined ICD-10 coding algorithms and categorized into the following groups: endocrine autoimmune diseases, rheumatologic disorders, connective tissue diseases, vasculitic disorders, neurologic autoimmune diseases, dermatologic autoimmune diseases, hematologic autoimmune diseases, gastrointestinal autoimmune diseases, and renal autoimmune diseases. The renal outcome labeled “IgA nephropathy” in the original analysis was defined using ICD-10 codes N02.8, N02.9, and N03.8.

### 2.4. Covariates and Confounders

Several potential covariates and confounders measured at birth or during early infancy that might influence the study outcomes were identified from diagnoses recorded during the birth admission or neonatal period in the NHIS database. These variables included socioeconomic and perinatal health factors: urban or rural residence at birth, household income level at birth, prematurity or fetal growth abnormalities, and neonatal hemorrhagic disorders.

### 2.5. Statistical Analysis

Baseline characteristics were summarized using descriptive statistics. Incidence rates were calculated as the number of events per 10,000 person-years (PYs).

Kaplan–Meier methods were used to estimate cumulative incidence of autoimmune diseases. Differences between groups were evaluated using the log-rank test.

Multivariable Cox proportional hazards regression models were used to estimate hazard ratios (HRs) and 95% confidence intervals (CIs). Adjusted models included all predefined demographic and perinatal covariates. Subgroup analyses were conducted according to sex, age at APN diagnosis, household income, prematurity status, and neonatal comorbidities.

To assess the presence of a biological gradient, analyses were repeated according to APN recurrence status. Children were categorized as having either a single APN episode or recurrent APN. Sensitivity analyses were performed using alternative lag periods of 0, 2, 3, and 5 years.

## 3. Results

### 3.1. Baseline Characteristics:

Table 1 summarizes the baseline characteristics of the cohorts. The study included 31,856 children in the APN-exposed cohort and 318,560 children in the matched unexposed cohort, with an equal sex distribution in both groups (50% girls and 50% boys). The median age at APN hospitalization was approximately 9 months. More than half of APN admissions occurred during the first year of life. Recurrent APN was identified in 668 children (2.1%), while the remaining children experienced a single APN episode.

### 3.2. Risk of Autoimmune Diseases:

By the end of follow-up, a total of 3839 children in the APN cohort and 23,520 children in the unexposed cohort were diagnosed with at least one autoimmune disease beyond the 1-year lag period. Because follow-up ended on 31 December 2017, the cohort was observed only through childhood and early adolescence; the maximum attainable age was approximately 12–15 years depending on birth year. Therefore, cumulative incidence estimates should not be interpreted as lifetime or adult risk. Kaplan–Meier analysis showed that the cumulative incidence of any autoimmune disease was 12.1% in APN-exposed children compared with 7.4% in unexposed children at the end of available follow-up (Figure 1). When individual autoimmune diseases were examined (Table 2), the increased risk was not uniform across all conditions; rather, certain disorders showed stronger associations with a history of APN.

**Figure 2:**
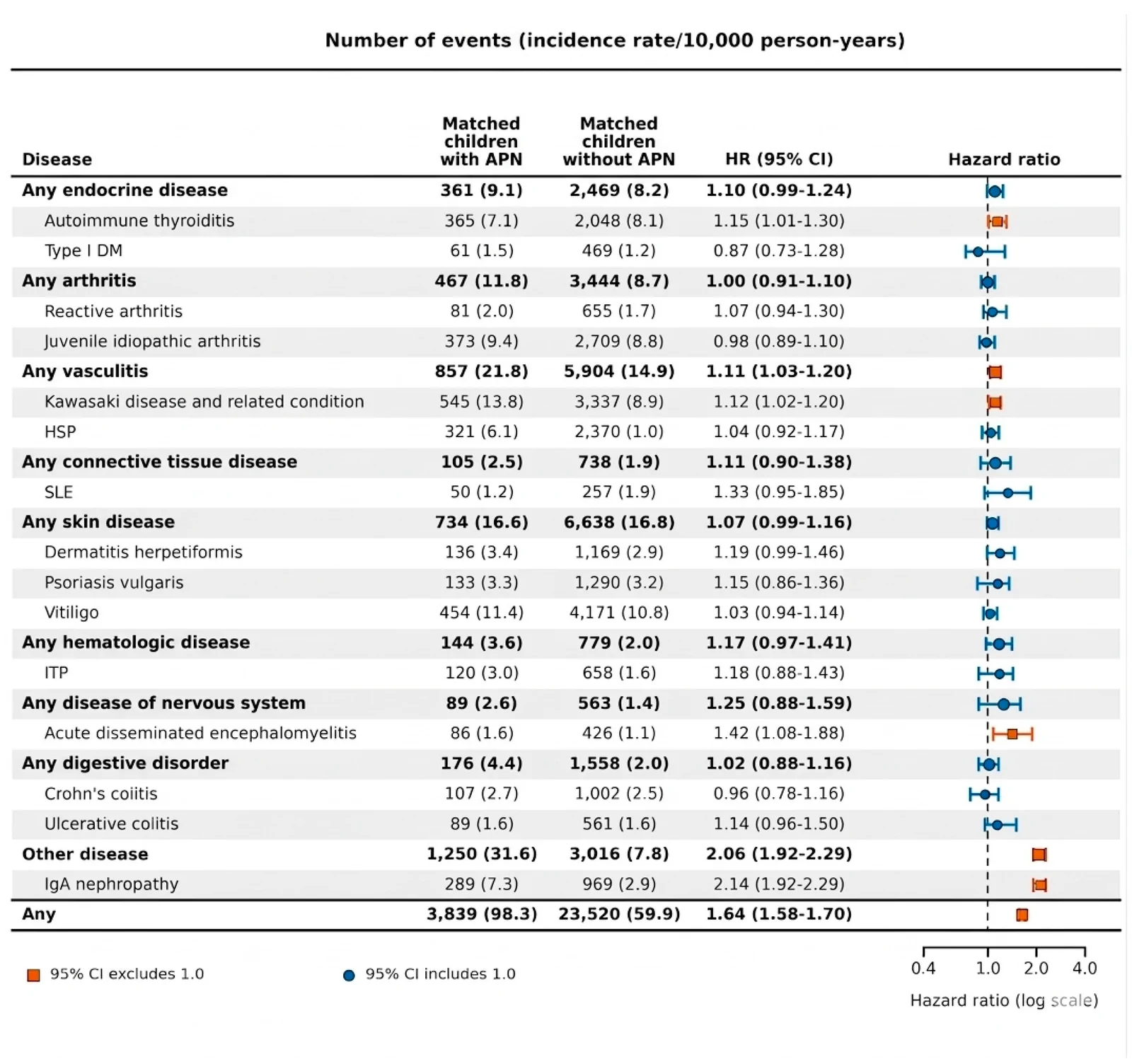
Forest plot of the incidence rate ratio (per 10,000 person-years) of APN exposed children. The APN group showed greater risk of IgA nephropathy (HR 2.14, 95% CI, 1.92–2.29) than the control group.

The incidence rates of autoimmune disease were 98.35 and 59.91 per 10,000 PYs in the APN and unexposed groups, respectively (Table 3). The adjusted hazard ratio (aHR) for developing any autoimmune disease in the APN cohort compared with controls was 1.641 (95% CI, 1.585–1.699; *p* < 0.001) (Table 3), indicating a higher risk of autoimmunity in the APN group. Figure 2 illustrates the adjusted hazard ratios for the composite outcome and for major categories of autoimmune diseases.

Endocrine Autoimmune Diseases: Autoimmune thyroiditis was more frequent in the APN group (incidence 7.66/10,000 PYs) compared with controls (5.13/10,000 PYs), corresponding to an aHR of 1.146 (95% CI 1.012–1.297). By age 15, 0.96% of the APN group developed autoimmune thyroiditis, compared to 0.64% of controls. In contrast, type 1 diabetes showed very low incidence in both groups and no significant difference was observed between two groups). Overall, combining all endocrine autoimmune diseases yielded an HR of 1.10 (95% CI 0.99–1.24) within the entire study cohort, which was not statistically significant.

**Arthritides:** Juvenile idiopathic arthritis (JIA), ankylosing spondylitis, and reactive arthritis as a group were evaluated in the study cohort. The incidence of JIA was around 11–12/10,000 PY in both groups, and the HR was 0.982 (0.879–1.097), indicating no significant difference. Reactive arthritis was slightly more frequent in APN-exposed children (HR 1.07) but with wide confidence intervals (CI > 1). Overall, no association was observed between APN and juvenile arthritis overall.

**Systemic Vasculitis:** Children with APN had a slightly higher incidence of vasculitic diseases. Kawasaki disease occurred more often in the APN group (HR 1.12, 95% CI 1.02–1.20). The APN group had a significantly higher risk of overall vasculitides (HR 1.11, 95% CI 1.03–1.19) compared with the control group. Although Behçet’s disease is rare in the pediatric population, the APN group had nine cases of Behçet’s disease, whereas 68 cases were observed in the control group (HR 4.86, 95% CI 1.99–11.87). These estimates should be interpreted cautiously because the event counts are small and the diagnosis is uncommon during childhood.

**Connective Tissue Diseases:** SLE occurred in 50 exposed vs. 257 unexposed children (HR 1.33, 95% CI 0.95–1.86), but the difference was not significant. Dermatomyositis/polymyositis and systemic sclerosis were very rare (no significant differences) in both groups. Overall, there was no clear association between APN and connective tissue autoimmune diseases, possibly due to limited cases by early adolescence.

**Dermatologic Autoimmune Diseases:** Vitiligo and psoriasis were the most common types. Vitiligo had an incidence of 14/10,000 PY in both groups by age 15 (HR 1.03, 95% CI 0.94–1.14), but showed no difference between the study groups. Psoriasis incidence was 8–9/10,000 PY (HR 1.15, 0.86–1.36) trended higher in APN children, but was not significant. Overall, there was no strong evidence of increased risk for most dermatological autoimmune conditions after APN.

**Hematologic Autoimmune Disorders:** This category included immune thrombocytopenic purpura (ITP) and autoimmune hemolytic anemia (AIHA). ITP occurred in 6.5/10,000 PY in APN children, whereas 5.5 in controls (HR 1.18, 95% CI 0.96–1.45). There were no recorded cases of pernicious anemia in this pediatric cohort. The combined hematologic autoimmunity outcome had HR 1.17, 95% CI 0.97–1.41, which was not statistically significant. Therefore, APN showed no association with the risk of developing ITP or related hematologic autoimmune conditions in childhood.

**Neurologic Autoimmune Diseases:** ADEM, an acute post-infectious demyelinating condition, showed significantly higher rate in APN children. There were 66 ADEM cases in the APN group (incidence 1.65/10,000 PY) and 426 in controls (1.07/10,000 PY), which yielded HR 1.42, 95% CI 1.08–1.88. The category of “any nervous system autoimmune disorder” had an HR 1.25 (95% CI, 0.88–1.59), indicating a higher risk of neurologic autoimmunity in the APN group, driven largely by ADEM cases.

**Digestive Autoimmune Diseases:** By the end of follow-up, the incidence of inflammatory bowel disease (IBD) was beginning to increase but remained relatively low, as the oldest subjects were in mid-adolescence. Crohn’s disease had HR 0.96 (95% CI 0.78–1.116), and ulcerative colitis HR 1.14 (95% CI 0.96–1.50), neither differing significantly between groups. The combined “any digestive autoimmune disease” outcome yielded HR 1.02 (95% CI 0.87–1.17), indicating no increase in pediatric IBD or other GI autoimmunity following APN.

**Other Autoimmune Disorders:** In this category, acute rheumatic fever (ARF), an autoimmune sequela of streptococcal throat infection, was less frequent in the APN-exposed group. The control group had 142 ARF cases (incidence, 0.36/10,000 PY) compared with 28 cases in the APN group (incidence, 0.70/10,000 PY); however, the reported HR of 0.59 is internally inconsistent with these crude counts and should not be interpreted until the source analysis is validated. The renal outcome previously labeled IgA nephropathy comprised 1223 exposed and 2881 unexposed cases under the N02.8/N02.9/N03.8 definition, corresponding to HR 2.14 (95% CI 1.92–2.29). Because these codes are not specific for biopsy-confirmed IgA nephropathy, the result should be interpreted as an association with the coded nephritic-syndrome outcome rather than definitive IgAN.

Overall, children with a history of APN had an increased risk of several autoimmune diseases, although the magnitude of risk varied across outcomes. However, the renal outcome should not be interpreted as biopsy-confirmed IgA nephropathy, as the ICD-10 codes used were not specific to this diagnosis.

### 3.3. Stratified Analysis

Table 3 presents subgroup analyses of the association between APN and the composite outcome of “any autoimmune disease.”

**By Sex:** Both girls and boys with APN had higher autoimmune risk than their counterparts without APN. The risk was slightly greater in females (HR 1.68, 95% CI 1.60–1.77) than in males (HR 1.61, 95% CI 1.53–1.69). The absolute incidence difference was slightly higher in girls (41.13/10,000 PY) compared with boys (36.08/10,000 PY). Thus, while female children generally have higher baseline rates of autoimmune conditions with a larger HR, the APN effect was present in both sexes.

**By Age at APN Index:** Children in the APN-exposed cohort were stratified by age at their first APN diagnosis: infants aged ≤6 months and children aged 7–24 months. The ≤6-month group comprised 18,984 children and had an HR of 1.426, whereas the 7–24-month group comprised 8130 children and had an HR of 1.607. A separate ≥25-month category was not present in the available subgroup table and should not be described without recalculation from the source dataset.

**By Birth Cohort:** The cohort was stratified by birth year (2002–2003 and 2004–2005) to evaluate whether birth cohort influenced the association with autoimmunity. The HR for autoimmunity was 1.67 (95% CI, 1.59–1.76) in the 2002–2003 birth cohort and 1.62 (95% CI, 1.54–1.70) in the 2004–2005 cohort, indicating no substantial cohort effect or time trend in the association between the groups.

**By Region and Socioeconomic Status:** Children from the Seoul metropolitan area had an HR of 1.605 (95% CI 1.53–1.68), whereas those from rural areas had an HR of 1.664 (95% CI 1.49–1.86); however, there was no clear urban–rural pattern. Regarding income of the cohort (above median vs. below median), there was no significant interaction between the study groups. Overall, the link between APN and autoimmunity is not confined to any particular demographic or socioeconomic factors.

**By Perinatal Health Status:** Children without any perinatal issues had a higher HR (1.69) compared with the ones with perinatal problems had a HR of 1.50. Among the 59 APN exposed preterm infants, the HR for autoimmunity was 2.01 (95% CI 1.49–2.71), whereas in full-term infants it was 1.64 (95% CI 1.58–1.69). Similar patterns were seen for infants with birth trauma, respiratory distress, or neonatal infection. The congenital-malformation subgroup in Table 3 represents claims for malformations that did not meet the predefined exclusion criterion for major congenital/chromosomal anomalies; this distinction has been clarified.

### 3.4. Dose–Response Analysis

The effect of more severe or repeated APN infections on autoimmune risk was evaluated and the results are presented in Table 4. Among the 31,856 exposed children, 31,188 had exactly one APN hospitalization, and 668 had recurrent APN (≥2 hospitalizations). Children with multiple APN episodes had a markedly higher rate of autoimmune outcomes: incidence 153.7/10,000 PY, compared to 97.3/10,000 PY in those with one APN episode. The adjusted HR for any autoimmune disease in the recurrent-APN group (vs. controls) was 2.58 (95% CI 2.16–3.13), compared with 1.62 (95% CI 1.57–1.68) for a single APN episode. This approximately 2.6-fold hazard in recurrent APN versus 1.6-fold hazard after a single episode is one of the strongest pieces of evidence supporting a possible biological gradient; however, recurrence may also mark children with greater healthcare utilization or underlying urinary tract abnormalities.

The impact of hospitalization duration as a proxy for infection severity was also evaluated. (Table 5) The APN cohort was categorized into two groups by length of initial hospital stay: short stays (≤3 days, which included those who had a single day or overnight hospitalization for disease evaluation) and longer stays (≥4 days; median stay 6 days). Both short and longer stay groups had elevated autoimmune risk relative to controls, with no significant difference between them. Specifically, children with shorter hospital stays had an adjusted HR of 1.67 (95% CI 1.61–1.73) for any autoimmune disease, while those with longer stays had HR 1.59 (95% CI 1.54–1.66).

### 3.5. Sensitivity Analyses (Lag Window)

In the primary analysis, autoimmune outcomes occurring within 1 year after the index date were excluded to reduce the potential for reverse causation. Additional sensitivity analyses used lag periods of 0, 2, 3, and 5 years; the results are presented in Table 6. The dose–response and coding-specific analyses indicate that more definitively severe infections confer higher risk, supporting a biological gradient. The persistence of association across various subgroups and lag times suggests that the relationship is not explained by a specific confounder or bias. While the magnitude of the association diminishes over time, a significant elevation in autoimmune risk remains even in the long term.

## 4. Discussion

In this large population-based cohort study, children who were hospitalized for APN had a significantly increased risk of developing autoimmune diseases later in childhood and adolescence compared to matched children without APN. The relative increase in risk was substantial, translating to an excess of about 3–4 additional cases of autoimmune disease per 1000 child-years of follow-up attributable to APN; however, this absolute estimate is subject to the same surveillance and outcome-misclassification limitations as the primary association. The magnitude of the association observed in the present study is greater than that reported in the Swedish population-based cohort of stress-related disorders, which reported an HR of 1.36; importantly, that Swedish cohort was predominantly adult, with a median age of 41 years at exposure, and therefore is not directly comparable with this pediatric cohort [8].

According to a previous Korean pediatric inpatient study [9], *E. coli* has consistently been the predominant urinary pathogen, accounting for approximately 81–83% of isolates in earlier cohorts, followed by Klebsiella pneumoniae. More recent multicenter data in young children also identify *E. coli* as the predominant organism, although antimicrobial resistance patterns require continued surveillance [10]. In addition, the current study outcomes align with prior evidence suggesting that early-life infections can have immunologic consequences. Specifically, a recent Korean study focusing on *E. coli* infections reported a smaller but significant increase (adjusted HR 1.25) in autoimmunity risk [11]. IgAN is generally believed to be triggered by mucosal immune responses post infections that leads to abnormal IgA deposition in the kidney [12]. The current study results showed HR 2.14 providing epidemiological support for this pathogenesis of IgAN, suggesting a severe UTI in early childhood might initiate immune dysregulation that manifests years later as IgAN. However, the present study cannot determine whether the observed association is attributable to a specific pathogen, to the systemic inflammatory response of APN, to antibiotic exposure, or to shared susceptibility. The association with the renal outcome should be interpreted cautiously because the ICD-10 codes used do not establish biopsy-confirmed IgA nephropathy.

Another notable association was ADEM, an autoimmune demyelinating disorder of the central nervous system that typically follows viral infections or vaccination [13], and children with APN exhibited a 40% higher hazard of developing this condition. Severe bacterial infection-induced systemic inflammation or blood–brain barrier disruption may increase susceptibility to subsequent ADEM [14]. Thus, a shared underlying immune dysregulation may contribute to the pathogenesis of both APN and ADEM.

A moderate increase in the risk of autoimmune thyroiditis, one of the most prevalent pediatric and adolescent autoimmune conditions [15], was also observed in this study. Given that thyroid autoimmunity is associated with specific infections and non-specific immune activation [16], the febrile inflammatory response in APN may accelerate thyroid autoimmunity in susceptible individuals.

The current study results showed no significant association between APN and type 1 diabetes or juvenile idiopathic arthritis, two major pediatric autoimmune conditions. This suggests that not all autoimmune diseases are influenced by early UTIs in the same way. Type 1 diabetes has been predominantly associated with viral pathogens, such as enteroviruses, rather than bacterial UTIs and this distinct etiology likely accounts for the lack of observed association in the present study [16]. Juvenile arthritis triggers are less well established [17], but the current study results indicate that APN does not appreciably raise JIA risk.

The current study results presented fivefold increase in Behçet’s disease risk within the APN cohort, although based on a limited number of cases, is noteworthy given that this vasculitis is hypothesized to be triggered by infectious agents in genetically predisposed individuals such as human leukocyte antigen (HLA)-B51 carriers [16]. The pediatric age range of this study cohort precedes the typical onset of Behçet’s disease [18].

One unexpected inverse association was the reduced incidence of ARF in children with APN. ARF is a classic post-infectious autoimmune condition caused by untreated streptococcal throat infections [19]. The lower ARF risk in the APN cohort may have several explanations. Several factors may account for the diminished risk of ARF within the APN cohort. Patients with a history of APN may have increased healthcare utilization, leading to a higher probability of prompt antibiotic intervention for subsequent febrile illnesses, which is a form of surveillance bias that may effectively prevent ARF. Importantly, the same surveillance mechanism could operate in the opposite direction for other outcomes: children with previous APN may have more frequent medical contacts and urinalyses, increasing the probability of detecting autoimmune conditions that might otherwise remain undiagnosed. Thus, surveillance bias could potentially contribute both to apparent positive associations and to the apparent inverse association for ARF. Furthermore, immune priming by Gram-negative pathogens, such as *E. coli*, may theoretically down-regulate autoimmune responses to subsequent Gram-positive infections [20]; however, this mechanism remains speculative. These findings suggest that early infectious exposures and associated clinical management may confer protective benefits against specific sequelae while increasing risk for others, underscoring the complex nature of immune-environment interactions.

The subgroup analyses showed that the association between APN and autoimmunity was present across several demographic and clinical subgroups. However, these findings do not exclude residual confounding by healthcare utilization. In particular, the substantially higher number of admissions and outpatient visits among APN-exposed children raises the possibility of differential ascertainment of autoimmune diseases. An active-comparator analysis using children hospitalized for other serious infections would be an important next step to separate the effect of APN itself from the effect of hospitalization, acute infection, and increased medical surveillance.

Regarding the age of first APN episode, children diagnosed with APN during the toddler years exhibited a higher relative risk for subsequent autoimmune sequelae than those diagnosed during the neonatal period. This observation may be attributed to the possibility that infants experiencing severe UTIs in early infancy accompany with underlying anatomical anomalies, such as vesicoureteral reflux, or primary immunodeficiencies. Such factors may independently increase baseline autoimmune susceptibility, thereby elevating the risk within the control cohort and attenuating the observed relative risk. In addition, APN in a previously healthy toddler might be a more random event, where subsequent autoimmunity is more readily identifiable against a lower baseline risk. Alternatively, the immune system in older infants may exhibit distinct responses to APN, potentially through the generation of autoreactive antibodies or memory cells that contribute to later autoimmune sequelae [20,21]. Regardless of these mechanistic nuances, APN at any stage of childhood appears to confer an elevated risk for autoimmune disorders.

The current study demonstrated a strong association between recurrent APN and subsequent autoimmune outcomes. The adjusted HR was 1.622 after a single APN episode and 2.579 after recurrent APN, suggesting a potential dose–response relationship. The observed dose–response relationship satisfies Hill’s criterion of biological gradient, indicating that repeated episodes of APN may cumulatively prime the immune system toward autoimmunity [22]. This process may be mediated by epitope spreading or microbial alterations [23]. Because risk was associated with recurrence but not with the length of stay, the current study data suggest that the occurrence of infection and the associated immune activation serves as the primary driver of subsequent autoimmunity, rather than the duration of the acute episode. Nevertheless, recurrent APN may also identify children with vesicoureteral reflux, other urinary tract abnormalities, immune susceptibility, or more intensive healthcare exposure. Therefore, the observed gradient supports but does not prove a definite biological dose–response mechanism.

Several hypotheses could explain an association between APN and later autoimmune disease, including molecular mimicry, bystander activation, epitope spreading, and disruption of immune regulatory networks. However, because the present database contains no microbiological or immunologic measurements, these mechanisms remain hypotheses rather than demonstrated pathways. Nevertheless, molecular mimicry can be one of the possible explanations: components of uropathogenic bacteria, including *E. coli* antigens, might share structural similarities with host tissues, leading to cross-reactive immune responses [24]. For example, *E. coli* infection has been implicated in autoimmunity of the liver (primary biliary cholangitis) through molecular mimicry with the mitochondrial autoantigen PDC-E2 [25]. Similar processes might occur with renal antigens in IgAN or vascular endothelium in Kawasaki disease [26,27]. Another possible pathway is bystander activation, wherein the intense systemic inflammation and cytokine release associated with APN trigger dormant self-reactive lymphocytes. Concurrently, tissue damage from renal parenchymal injury may release sequestered self-antigens, thereby compromising immune tolerance [28]. Epitope spreading may further contribute to disease pathogenesis, wherein the primary immune response to an infectious agent progressively expands to encompass host antigens. This process is potentially exacerbated when tissue injury exposes sequestered host proteins to the immune system, facilitating an autoreactive response [29]. Furthermore, severe infection may modulate immune regulatory networks, potentially disrupting the homeostatic balance between regulatory T-cells and effector T-cells [30]. Moreover, genetic predisposition could be considered; children predisposed to autoimmune diseases may exhibit increased susceptibility to severe infections. Specifically, genetic variants modulating immune function can simultaneously influence infective susceptibility and autoimmunity, positioning APN as a potential marker of underlying immune dysregulation [31]. However, the observed dose–response relationship and specificity toward distinct autoimmune outcomes suggest that infection exerts an independent pathogenic effect, rather than serving only as an epiphenomenon.

Pediatric patients with APN frequently undergo early-life exposure to broad-spectrum antibiotics, which may disrupt nascent gut microbiota composition. Emerging evidence indicates that early-life dysbiosis modulates immune system maturation, potentially escalating the risk of autoimmune pathologies such as type 1 diabetes, allergies, and other autoimmune disorders [32]. Broad-spectrum antibiotic exposure may modulate autoimmune risk through bidirectional mechanisms: escalating risk via microbial depletion and pro-inflammatory dysbiosis, or mitigating it through the eradication of potential arthritogenic or immunogenic bacterial triggers [32]. As the current study presents an elevated incidence of most autoimmune phenotypes, any secondary microbiota-mediated effects of antibiotic exposure must logically align with this positive association. Some studies suggests that early-life antibiotic-induced dysbiosis drives autoimmunity in animal models via the disruption of immune cell homeostasis [33]. Thus, the present study cannot distinguish the effect of infection from the effect of antibiotic treatment because detailed antimicrobial exposure, duration, prophylaxis, and microbiome data were unavailable.

This study possesses several notable strengths. The nationwide design minimized selection bias and ensured broad population representativeness. The large sample size enabled evaluation of relatively uncommon autoimmune diseases that would be difficult to study in smaller cohorts. Longitudinal follow-up permitted assessment of autoimmune outcomes through childhood and early adolescence. Furthermore, the recurrence analysis provided evidence of a possible dose–response pattern.

Several limitations should be acknowledged. First, the observational design precludes definitive causal inference and residual confounding remains possible. Second, disease definitions relied on administrative claims data and may be subject to diagnostic misclassification. Third, APN was defined from inpatient ICD-10 coding without urine culture, organism identification, antimicrobial susceptibility, urinalysis, inflammatory-marker, DMSA, or ultrasonographic information; therefore, exposure misclassification and misdiagnosis of febrile illnesses are possible. Fourth, antibiotic treatment is intrinsically linked to the APN exposure in hospitalized children, and the study cannot disentangle pathogen-related effects from antibiotic-related microbiome effects. Fifth, APN-exposed children had substantially more healthcare encounters, creating potential detection/surveillance bias. Sixth, unmeasured urinary tract anomalies such as vesicoureteral reflux and CAKUT may confound both recurrent APN and subsequent autoimmune outcomes. Seventh, the renal outcome was based on nonspecific ICD-10 codes and cannot establish biopsy-confirmed IgA nephropathy. Eighth, active-comparator analyses using children hospitalized for other infections were not available in the present dataset. Finally, follow-up was limited to childhood and early adolescence and generalizability beyond the Korean population, including populations with different background rates of Kawasaki disease, is uncertain.

Given these limitations, the observed associations should be considered hypothesis-generating rather than evidence that APN causes autoimmune disease. The absolute differences were modest for most individual outcomes and were driven in part by the composite outcome. Future studies with microbiological characterization, detailed antibiotic exposure, imaging and urinary-tract phenotyping, active infection comparators, and prospective follow-up are needed to determine whether specific pathogens or treatment exposures contribute to autoimmune risk.

## 5. Conclusions

In this nationwide population-based cohort study, childhood hospitalization for APN was associated with increased risk of subsequent autoimmune disease during childhood and early adolescence. The stronger association with recurrent APN supports a potential dose–response relationship, but attenuation with longer lag periods and the possibility of surveillance bias, exposure misclassification, urinary tract anomalies, and antibiotic-related effects limit causal interpretation. These findings provide epidemiologic evidence linking severe childhood bacterial infection with later autoimmune disease susceptibility and underscore the need for mechanistic studies investigating the long-term immunologic consequences of pediatric APN.

## Figures and Tables

**Table 1:** Baseline characteristics.

Characteristics	Population-Matched Cohort	
	Children with APN	Children Without APN
**Number of participants**	31,856	318,560
**Follow-up time, mean ± SD (range), months**	154 ± 37.8 (151–176)	160 ± 28.5 (152–177)
**Sex, n (%)**		
**Boy**	15,949 (50.1)	159,490 (50.1)
**Girl**	15,907 (49.9)	159,070 (49.9)
**Age at index date, mean ± SD (range), month**	25.4 ± 35.9 (4–40)	28.2 ± 37.8 (4–40)
**Birth residence**		
**Seoul or Metropolitan**	16,828 (52.8)	167,526 (52.6)
**Suburban city**	11,760 (36.9)	118,807 (37.3)
**Rural**	3268 (10.3)	32,171 (10.1)
**Income median**		
**Above median**	15,760 (49.5)	157,834 (49.6)
**Below median**	16,096 (50.5)	160,726 (50.4)
**By calendar year at birth date, n (%)**		
**Year 2002–2003**	14,510 (45.5)	145,100 (45.5)
**Year 2004–2005**	17,346 (54.5)	173,460 (54.5)
**^a^** **Number of admission during the first year after study entry, n (%)**		
**0 times (no)**	21,985 (69.0)	276,479 (86.8)
**≥1 times (yes)**	9871 (31.0)	42,081 (13.2)
**No. of outpatient visit during the first year after study entry, n (%)**		
**<Median (<14 visits)**	13,333 (41.9)	168,345 (52.9)
**≥Median (≥14 visits)**	18,523 (58.1)	150,215 (47.1)
**Perinatal status, n (%)**		
**Any perinatal status**		
**Yes**	5279 (16.6)	38,852 (12.2)
**No**	26,577 (83.4)	279,708 (87.8)
**Disorders related to length of gestation and fetal growth**		
**Yes**	375 (1.2)	2781 (0.9)
**No**	31,481 (98.8)	315,779 (99.1)
**Birth Injury**		
**Yes**	77 (0.2)	629 (0.2)
**No**	31,779 (99.8)	317,931 (99.8)
**Respiratory and cardiovascular disorders specific to the perinatal period**		
**Yes**	463 (1.5)	3042 (0.9)
**No**	31,393 (98.5)	315,518 (99.1)
**Infections specific to the perinatal period**		
**Yes**	2827 (8.9)	18,118 (5.7)
**No**	29,029 (91.1)	300,442 (94.3)
**Hemorrhagic disorders of fetus and newborn**		
**Yes**	2788 (8.8)	22,693 (7.1)
**No**	29,068 (91.2)	295,867 (92.9)
**Systemic steroid prescription exposure index date (on admission status)**		
**Yes**	1708 (5.4)	-
**No**	30,148 (94.6)	-
**Days with admission**		
**<Median (<7 days)**	16,861 (52.9)	-
**≥Median (≥7 days)**	14,995 (47.1)	-
**Intensive care during an admission status after index date**		
**Yes**	275 (0.9)	
**No**	31,581 (99.1)	
**Urinary tract infection frequency**		
**1 event**	31,188 (97.9)	
**≥2 events**	668 (2.1)	

^a^ Removed the first month after study entry from this calculation. APN, acute pyelonephritis; SD, standard deviation.

**Table 2:** Disease-Specific Associations with Autoimmune Diseases (After 12 months from Index date).

					Matched Children with APN			Matched Children Without APN			Rate Difference			Hazard Ratio		
					No. of AID Cases	No. of APY × 10,000	IR/10,000 Person-Years	No. of AID Cases	No. of APY ×10,000	IR/10,000 Person-Years	ARD/10,000 Person-Years	95% CI Lower	95% CI Upper	Adjust HR	95% CI Lower	95% CI Upper
**Any disease**					3839	39	98.35	23,520	392.6	59.91	38.449	35.245	41.653	1.641	1.585	1.699
**Endocrine**	**Autoimmune thyroiditis [E03.5, E03.9, E05.0, E05.5, E05.9, E06.3, E06.5]**				305	39.8	7.66	2048	398.8	5.13	2.527	1.6348	3.415	1.146	1.012	1.297
	**Type I DM [E10]**				61	39.9	1.53	469	399.7	1.17	0.354	−0.044	0.752	**0.** **8** **7**	0.733	1.283
	**Addison’s disease [E27.1, E27.2]**				4	40	0.1	5	400	0.01	0.088	−0.011	0.186	-	-	-
	**Autoimmune polyglandular syndrome [E31.0]**				0	40	0	7	400	0.02	−0.018	−0.031	−0.005	-	-	-
	**Any endocrine disease**				361	39.8	9.07	2469	398.6	6.19	2.880	1.913	3.848	1.105	0.987	1.238
**Arthritis**	**Reactive arthritis (Reiter’s syndrome) [M02.3, M02.8, M02.9]**				81	39.9	2.03	655	399.6	1.64	0.389	−0.070	0.849	1.068	0.839	1.36
	**Juvenile idiopathic arthritis [M05, M06, M08.0, M08.1, M08.2, M08.3, M08.4]**				373	39.8	9.37	2709	398.5	6.8	2.576	1.590	3.561	0.982	0.879	1.097
	**Ankylosing spondylitis [M45]**				28	40	0.7	177	399.9	0.44	0.258	−0.010	0.525	0.975	0.633	1.502
	**Any arthritis**				467	39.7	11.75	3444	398.1	8.65	3.103	1.998	4.207	1.001	0.907	1.105
**Vasculitis**	**Polyarteritis nodosa and related condition (Incl. Kawasaki, Churg-Strauss syndrome) [M30]**				545	39.6	13.77	**3** **3** **37**	396.8	8.91	4.856	3.663	6.049	1.122	1.023	**1.2** **03**
	**Thrombotic microangiopathy [M31.1]**				0	40	0	1	400	0	−0.003	−0.008	0.002	-	-	-
	**Granulomatosis with polyangiitis [M31.3]**				0	40	0	3	400	0.01	−0.008	−0.016	0.001	-	-	-
	**Microscopic polyangiitis [M31.7]**				0	40	0	1	400	0	−0.003	−0.007	0.002	-	-	-
	**Henoch-Schönlein purpura [D690]**				321	39.7	8.08	2370	398	5.95	2.124	1.208	3.039	1.038	0.921	1.170
	**Giant cell arteritis/Polymyalgia rheumatica [M35.3, M31.5, M31.6]**				2	40	0.05	28	400	0.07	−0.020	−0.094	0.054	12.877	1.148	144.441
	**Any Vasculitis**				857	39.3	21.78	5904	394.9	14.95	6.833	5.326	8.341	1.112	1.034	1.196
**Connective**	**Systemic lupus erythematosus [M32]**				**18**	40	1.25	**107**	399.8	0.64	0.609	0.253	0.964	1.329	0.953	1.855
	**Polymyositis/dermatomyositis [M33.0, M33.1, M33.2, M33.9]**				20	40	0.5	168	399.9	0.42	0.080	−0.148	0.309	1.172	0.673	2.042
	**Systematic sclerosis [M34]**				2	40	0.05	12	400	0.03	0.020	−0.051	0.091	-	-	-
	**Sjögren’s syndrome [M35.0]**				25	40	0.63	247	399.8	0.62	0.008	−0.249	0.265	0.857	0.554	1.326
	**Mixed connective tissue disease [M35.1]**				1	40	0.03	10	400	0.03	0.008	−0.249	0.265	-	-	-
	**Behcet’s syndrome [M35.2]**				**4**	40	0.23	**1**	399.9	0.17	0.055	−0.097	2077	4.86	1.99	11.873
	**Any connective tissue disorders**				105	39.9	2.63	738	399.5	1.85	-	-	-	1.112	0.897	1.379
**Skin**	**Pemphigus vulgaris [L10.0]**				3	40	0.08	22	399.9	0.06	0.020	−0.068	0.108	0.843	0.083	8.605
	**Bullous pemphigoid [L12]**				7	40	0.18	86	399.9	0.22	−0.040	−0.177	0.098	0.931	0.408	2.125
	**Dermatitis herpetiformis [L13.0]**				136	39.9	3.41	1169	399	2.93	0.482	−0.116	1.079	1.186	0.988	**1.4** **5** **5**
	**Psoriasis vulgaris [L40]**				133	39.9	3.33	1290	399.1	3.23	0.102	−0.491	0.695	1.148	**0.** **8** **57**	**1.3** **64**
	**Alopecia areata [L64]**				9	40	0.23	43	400	0.11	0.118	−0.033	0.268	2.12	0.683	6.578
	**Vitiligo [L80]**				454	39.6	11.45	4171	397	10.51	0.943	−0.157	2.044	1.032	0.935	1.139
	**Any skin disease**				734	39.4	18.61	6638	395.2	16.8	1.817	0.411	3.223	1.072	0.992	1.159
**Hematologic**	**Autoimmune hemolytic anemia [D59.0, D59.1]**				23	40	0.58	116	399.9	0.29	0.285	0.0443	0.526	0.908	0.522	1.579
	**Immune thrombocytopenic purpura [D693]**				120	39.9	3.01	658	399.4	1.65	1.360	0.808	1.913	1.177	**0.** **883**	**1.4** **2** **7**
	**Pernicious anemia [D51.0]**				1	40	0.03	8	400	0.02	0.005	−0.046	0.056	-	-	-
	**Any hematologic disease**				144	39.9	3.61	779	399.4	1.95	0.005	−0.046	0.056	1.169	0.969	1.410
**Nervous**	**Acute disseminated encephalomyelitis [G04]**				**8** **6**	39.9	1.65	426	399.7	1.07	0.587	0.175	0.998	1.417	1.079	**1.8** **8** **4**
	**Anti-NMDA receptor encephalitis [G13.1]**				0	40	0	0	400	0	-	-	-	-	-	-
	**Multiple sclerosis [G35]**				5	40	0.13	26	400	0.07	0.060	−0.052	0.173	0.928	0.234	3.682
	**Neuromyelitis optica and ADEM [G36]**				1	40	0.03	21	400	0.05	−0.027	−0.081	0.0264	-	-	-
	**Guillain-Barre syndrome [G61.0, G61.1, G61.8, G61.9]**				10	40	0.25	70	399.9	0.18	0.075	−0.085	0.236	1.407	0.606	3.27
	**Myasthenia gravis [G70.0]**				7	40	0.18	45	399.9	0.11	0.063	−0.071	0.196	2.119	0.732	6.133
	**Any disease of nervous system**				89	39.9	2.23	563	399.6	1.41	0.819	0.342	1.297	**1.25** **3**	**0.** **881**	1.592
**Digestive**	**Primary biliary cirrhosis [K74.3]**				1	40	0.03	5	400	0.01	0.0125	−0.038	0.063	-	-	-
	**Crohn’s disease [K50]**				107	39.9	2.68	1002	399.4	2.51	0.172	−0.359	0.703	0.964	0.783	**1.1** **62**
	**Ulcerative colitis [K51]**				**89**	39.9	1.65	561	399.6	1.4	0.249	−0.167	0.664	**1.1** **43**	**0.** **9** **6** **4**	1.496
	**Coeliac disease [K90.0]**				2	40	0.05	11	400	0.03	0.023	−0.049	0.094	-	-	-
	**Any digestive disorder**				176	39.9	4.41	1558	399	3.9	0.509	−0.171	1.189	1.016	0.865	**1.1** **6** **4**
**Other**	**Acute rheumatic fever and chorea [I00, I01.0, I01.1, I01.2, I01.8, I01.9, I02.0, I02.9]**				28	40	**0.** **7**	142	399.8	**0.** **36**	0.346	0.0795	0.612	0.59	0.369	0.944
	**Sarcoidosis [D86]**				0	40	0	0	400	0	-	-	-	-	-	-
	**IgA nephropathy [N02.8, N02.9, N03.8]**				**289**	39.6	30.87	**969**	397.9	7.24	23.635	21.885	25.386	2.138	**1.9** **2** **4**	2.293
	**Any Other disorders**				1250	39.6	31.57	3016	397.8	7.58	23.991	22.220	25.763	2.055	1.919	2.202

APN, acute pyelonephritis; AID, autoimmune disease; APY, accumulated person-years; IR, incidence rate; ARD, absolute rate difference.

**Table 3:** Subgroup Analyses.

	Matched Children with APN				Matched Children Without APN				Rate Difference			Hazard Ratio			
	No. of Patients	No. of AID Cases	No. of APY × 10,000	IR/10,000 Person-Years	No. of Patients	No. of AID Cases	No. of APY × 10,000	IR/10,000 Person-Years	ARD/10,000 Person-Years	95% CI Lower	95% CI Upper	Adjusted HR	95% CI Lower	95% CI Upper	*p*-Value
**All**	31,856	3839	39	98.35	318,560	23,520	392.6	59.91	38.4489	35.2446	41.6531	1.641	1.585	1.699	-
**By sex**															
**Female**	15,907	1810	18.1	100.12	15,9070	10,738	182.1	58.98	41.1301	36.39	45.88	1.684	**1.6** **01**	1.771	0.051
**Male**	15,949	2029	21	96.82	15,9490	12,782	210.5	60.72	36.0842	31.74	40.43	1.607	1.533	1.686	
**Age group at index date, m**															
**≤6 month**	18,984	2388	26.4	90.56	17,7530	15,537	252.2	61.61	26.9028	22.63	31.18	1.426	1.356	1.5	0.007
**7–24 month**	8130	1044	10.7	97.13	82,860	6759	113	59.82	37.2968	31.24	43.36	1.607	1.503	1.717	
**By calendar year at birth date, No. (%)**															
**Year 2002–2003**	14,510	1841	16.7	110.39	145,100	11,151	169	65.98	44.3934	39.21	49.58	**1.67** **0**	1.588	1.756	0.434
**Year 2004–2005**	17,346	1998	22.4	89.37	173,460	12,369	223.6	55.33	34.031	29.99	38.07	1.616	1.54	1.695	
**Birth residence**															
**Seoul or Metropolitan**	16,828	2052	20.8	98.54	167,526	12,684	206.4	61.45	37.1056	32.71	41.50	1.605	1.531	1.683	0.249
**City**	11,760	1408	14.3	98.3	118,807	8571	146.5	58.52	39.7989	34.52	45.08	1.691	1.597	1.791	
**Rural**	3286	379	3.9	97.52	32,171	2265	39.7	57.12	40.3045	30.22	50.39	1.664	1.485	1.864	
**Income median**															
**<Median**	9284	1043	11.2	93.53	93,322	6593	114.9	57.39	36.1523	30.31	42.00	1.584	1.478	1.698	0.225
**≥Median**	22,572	2796	27.9	100.28	225,238	16,927	277.7	60.95	39.3327	35.50	43.16	1.66	1.595	1.728	
**Any perinatal status**															
**Yes**	7342	976	9.1	107.27	54,568	4899	67.5	72.6	34.6057	28.37	40.84	1.501	1.408	1.6	0.006
**No**	24,514	2863	29.9	95.64	263,992	18,621	325.1	57.28	38.8188	35.08	42.56	1.685	1.617	1.756	
**Disorders related to length of gestation and fetal growth**															
**Yes**	375	59	0.5	127.16	2781	216	3.4	63.04	65.2871	31.50	99.08	2.009	1.489	2.711	0.179
**No**	31,481	3780	38.6	98	315,779	23,304	389.2	59.88	38.1193	34.90	41.34	1.636	1.58	1.694	
**Birth Injury**															
**Yes**	77	10	0.1	104.62	629	50	0.8	62.73	87.5	9.64	165.36	2.808	1.561	5.054	0.118
**No**	31,779	3824	38.9	98.21	317,931	23,470	391.8	59.91	38.2963	35.09	41.50	1.638	1.582	1.696	
**Respiratory and cardiovascular disorders specific to the perinatal period**															
**Yes**	463	59	0.6	104.34	3042	237	3.7	63.41	40.1398	12.52	67.76	1.57	1.167	2.111	0.803
**No**	31,393	3780	38.5	98.26	315,518	23,283	388.8	59.88	38.3803	35.15	41.61	1.641	1.585	1.699	
**Infections specific to the perinatal period**															
**Yes**	2827	359	3.6	100.37	18,118	1505	22.6	66.65	33.6274	22.72	44.53	1.494	1.328	1.681	0.105
**No**	29,029	3480	35.5	98.15	300,442	22,015	370	59.5	38.6387	35.28	41.99	1.656	1.597	1.717	
**^@^** **Minor congenital malformations**															
**Yes**	4734	650	5.8	112.01	37,123	3503	45.7	76.63	35.4337	26.45	44.42	1.483	1.363	1.615	0.016
**No**	27,122	3189	33.2	95.97	281,437	20,017	346.9	57.71	38.2583	34.83	41.68	1.659	1.597	1.723	
**No. of admission during the first year after study entry**															
**Yes**	9871	1384	12.5	111.02	42,081	4635	55.8	82.99	27.9962	21.68	34.31	1.298	1.221	1.38	<0.001
**No**	21,985	2455	26.6	92.41	276,479	18,885	336.7	56.08	36.3139	32.57	40.06	1.638	1.57	1.71	
**No. of outpatient visit during the first year after study entry**															
**<Median**	11,658	1151	13.7	84.18	169,210	10,475	202.1	51.84	32.3605	27.40	37.33	1.585	1.49	1.686	0.504
**≥Median**	20,198	2688	25.4	105.99	149,350	13,045	190.5	68.47	37.5196	33.34	41.70	1.545	1.481	1.612	

^@^ Minor congenital anomaly includes non-syndromic renal anomalies such as mild isolated duplicated collecting systems.

**Table 4:** Recurrent APN and Biological Gradient (by number of APN episode).

	Matched Children with APN				Matched Children Without APN				Hazard Ratio			
	No. of Subject	No. of AID Cases	No. of APYs × 10,000	IR/10,000 Person-Years	No. Subject	No. of AID Cases	No. of APY × 10,000	IR/10,000 Person-Years	HR	95% CI Lower	95% CI Upper	*p*-Value
**No. of APN episodes**												
**1**	31,188	3724	38.3	97.27	318,560	23,520	392.6	59.91	1.622	1.566	1.68	<0.001
**≥2**	668	115	0.7	153.73					2.579	2.155	3.129	0.003

**Table 5:** APN and Biological Gradient (by hospitalization duration).

**Children with APN (Overall)**				**Children with APN (Hospitalized Cases for Less Than Median Days After APN Infection)**				**Hazard Ratio**			
**No. of Subject**	**No. of AID Cases**	**No. of APY × 10,000**	**IR/10,000 Person-Years**	**No. of Subject**	**No. of AID Cases**	**No. of APY × 10,000**	**IR/10,000 Person-Years**	**Adjusted HR**	**95% CI Lower**	**95% CI Upper**	***p*-Value**
**31,856**	23,520	392.6	59.91	**15,769**	1885	18.8	100.01	1.665	1.606	1.726	0.554
				**Children with APN** **(Hospitalized Cases for More Than Median Days After APN Infection)**				**Adjusted Hazard Ratio**			
				**No. of Subject**	**No. of AID Cases**	**No. of Accumulated Person-Years** **×** **10,000**	**Incidence Rate/10,000 Person-Years**	**HR**	**95% CI Lower**	**95% CI Upper**	***p*** **-Value**
				**16,087**	2131	22.3	95.53	1.597	1.539	1.657	0.593

Median hospitalization period for APN = 6 days.

**Table 6:** Sensitivity analyses across lag periods.

	Matched Children with APN				Matched Children Without APN				Rate Difference			Hazard Ratio			
Lag Period	No. of Subjects	No. of AID Cases	No. of APR × 10,000	IR/10,000 Person-Years	No. of Subjects	No. of AID Cases	No. of APR × 10,000	IR/10,000 Person-Years	Absolute Rate Difference/10,000 PY	95% CI Lower	95% CI Upper	Adjusted HR	95% CI Lower	95% CI Upper	*p*-Value
0-year	31,856	3839	41.4	84.8	318,560	23,520	420.1	51.61	35.105	32.058	38.152	1.581	1.530	1.634	<0.001
1-year	31,856	3839	39.0	98.35	318,560	23,520	392.6	59.91	38.448	35.244	41.653	1.641	1.585	1.699	<0.001
2-year	31,856	3839	33.3	115.38	318,560	23,520	348.2	67.55	13.132	10.279	15.984	1.208	1.155	1.264	<0.001
3-year	31,856	3839	30.3	126.68	318,560	23,520	317.3	74.12	11.281	8.377	14.184	1.182	1.126	1.241	0.001
5-year	31,856	3839	24.5	156.56	318,560	23,520	257.0	91.52	8.677	5.615	11.739	1.14	1.077	1.207	<0.001

## Data Availability

The data analyzed in this study are presented in the article, figures, and tables. Restrictions apply because the data were used under the terms of the current study and are not publicly available. The data may be requested from the Ministry of Health and Welfare, Republic of Korea, subject to applicable permissions.

## Supplementary Materials

The following supporting information can be downloaded at: https://www.mdpi.com/article/10.3390/pathogens15101096/s1, Table S1: Microbiological data availability for APN cases.

## Abbreviations

ADEM, acute disseminated encephalomyelitis; AID, autoimmune disease; APN, acute pyelonephritis; APY, accumulated person-years; ARD, absolute rate difference; CAKUT, congenital anomalies of the kidney and urinary tract; CI, confidence interval; GBS, Guillain–Barré syndrome; HR, hazard ratio; HSP, Henoch-Schönlein purpura; IgAN, IgA nephropathy; ITP, immune thrombocytopenic purpura; IR, incidence rate; NHIS, National Health Insurance Service; PY, person-years; SLE, systemic lupus erythematosus; UTI, urinary tract infect.
