# Supplementary material for: Long-Term Autoimmune Risk Following Pediatric Acute Pyelonephritis: A Nationwide Cohort Study
Source: Pathogens. 2026 Oct 21;15(10):1096. doi: 10.3390/pathogens15101096 (PMC13708077; doi:10.3390/pathogens15101096)
Supplement: Supplementary file 1 [file pathogens-15-01096-s001.zip › pathogens-4621155-supplementary.pdf]

SUPPLEMENTARY TABLE

Table S1. Microbiological data availability for APN cases

Variable	APN cohort (n=31,856)	Availability in NHIS claims data
Urine culture result	31,856	Not available in analytic dataset
Causative organism (E. coli)	31,856	Not available in analytic dataset
Other bacterial species	31,856	Not available in analytic dataset
Mixed infection	31,856	Not available in analytic dataset
Antimicrobial susceptibility	31,856	Not available in analytic dataset
Antibiotic agent/duration	31,856	Not available in analytic dataset
DMSA/renal ultrasonography	31,856	Not available in analytic dataset

APN; acute pyelonephritis, NHIS; National Health Insurance Service
